# Diagnostic value of combined detection of pepsinogen, gastrin-17, and ^13^C-urea breath test in Helicobacter pylori-associated chronic gastritis in children: a multivariate analysis

**DOI:** 10.3389/fped.2025.1696889

**Published:** 2025-12-15

**Authors:** Yiyun Gao, Chuangui Liu, Kaishi Ouyang, Siqi Li, Weilin Huang

**Affiliations:** 1 Department of Clinical Laboratory, Shunde District Hospital of Integrated Traditional Chinese and Western Medicine (Jun’an Hospital of Shunde District), Foshan, Guangdong, China; 2 Department of Dermatology, Shunde District Hospital of Integrated Traditional Chinese and Western Medicine (Jun’an Hospital of Shunde District), Foshan, Guangdong, China; 3 Department of Anesthesiology, Shunde Hospital of Guangzhou University of Chinese Medicine, Foshan, Guangdong, China

**Keywords:** gastrin-17, pepsinogen, ^13^C-urea breath test, chronic gastritis in children, multivariate logistic regression analysis

## Abstract

**Background:**

This study evaluated the diagnostic efficacy of combining pepsinogen (PG I/II), gastrin-17 (G-17), and ^13^C-urea breath test (13C-UBT) for chronic gastritis in children using logistic regression and receiver operating characteristic (ROC) analysis.

**Methods:**

Between June 2018 and August 2023, 65 children with chronic gastritis and 50 healthy controls participated. Inclusion criteria for both groups: Age 6–12 years, chronic gastritis confirmed by endoscopy and histopathology for the gastritis group, and absence of GI symptoms for the control group. Exclusion criteria: Severe cardiopulmonary dysfunction, gastroduodenal surgery, recent use of PPIs/antibiotics, and H. pylori eradication therapy. Serum levels of PG I, PG II, and G-17 were determined by ELISA, and 13C-UBT was used to detect Helicobacter pylori (Hp) infection.

**Results:**

Combined detection showed significantly higher positive rates than individual PG I/II or G-17 tests (*P* < 0.05), but similar to 13C-UBT alone. Chronic gastritis patients had elevated PG I, and G-17 levels compared to controls (*P* < 0.05). Multivariate analysis identified PG I (OR = 1.048, *P* = 0.014), G-17 (OR = 1.943, *P* < 0.001), and 13C-UBT positivity (OR = 17.417, *P* < 0.001) as significant predictors. ROC analysis revealed AUCs of 0.692 (PG I), 0.594 (PG II), 0.782 (G-17), 0.808 (13C-UBT), and 0.844 (combined), with sensitivity ≥90.8% and specificity >78%.

**Conclusion:**

The combined detection of PG I/II, G-17, and 13C-UBT shows strong diagnostic potential for Helicobacter pylori-associated chronic gastritis in children, offering enhanced diagnostic accuracy and a non-invasive tool for early screening and intervention.

## 1 Introduction

Chronic gastritis (CG) is a prevalent digestive system disorder characterized by prolonged inflammation of the gastric mucosa. It is primarily classified into superficial, atrophic, and specific forms of gastritis (1). The pathological manifestations include damage to the gastric mucosal epithelium, a reduction in glandular structures, and infiltration of inflammatory cells. Chronic gastritis arises from multiple factors, including *Helicobacter pylori* (Hp) infection, lifestyle choices (e.g., smoking, alcohol use), dietary patterns, and nonsteroidal anti-inflammatory drugs (NSAIDs) overuse (2, 3). Epidemiological data reveal a significant global prevalence of chronic gastritis, especially in middle-aged and elderly populations, with incidence rates rising with age. The clinical symptoms typically include epigastric discomfort, nausea, and loss of appetite, though some patients may remain asymptomatic. In recent years, the incidence of chronic gastritis in children has also risen, with regional incidence rates in China ranging between 45% and 80% due to variations in dietary patterns (4, 5). The gastric mucosa of children is comparatively fragile, making it more susceptible to damage and increasing the risk of gastritis. Chronic gastritis in children is often caused by Hp infection, which is particularly prevalent in younger children with immature immune systems, especially in the age of 10 age (6). Other etiologies, such as dietary factors, NSAIDs, and genetic predispositions, may also contribute to the development of chronic gastritis in pediatric populations. Early prevention and therapy in pediatric populations are essential because they can reduce the likelihood that serious gastrointestinal diseases will develop in adulthood.

Gastroscopy offers clinicians direct diagnostic evidence for chronic gastritis; however, challenges such as incomplete physiological development, low compliance, and safety concerns related to anesthesia restrict its application in pediatric populations (7, 8). Therefore, developing more practical diagnostic methods for chronic gastritis in pediatric patients is essential. Serological markers, including pepsinogen I (PG I) and pepsinogen II (PG II), indicate the condition of intestinal mucosa. A diminished PG I/PG II ratio (PGR) generally signifies atrophic gastritis, a disease intimately associated with later stomach carcinogenesis (9). Gastrin-17 (G-17), a precursor of gastrin, may signal disease progression of chronic gastritis toward pre-cancerous lesions when elevated (10). Furthermore, the non-invasive ^13^C-urea breath test (^13^C-UBT) holds significant value in diagnosing and managing Hp infection, a key contributor to chronic gastritis (11).

This study includes pediatric patients diagnosed with chronic gastritis from the gastroenterology department, alongside healthy children serving as controls. The analysis of PG I/II, G-17, and ^13^C-UBT results using various mathematical-statistical models aimed to assess the clinical utility of these established serological and breath-test markers for the screening and diagnosing of chronic gastritis in children.

## 2 Materials and methods

### 2.1 General information

This study is a retrospective analysis of children diagnosed with chronic gastritis. The study utilized previously collected data from the period between June 2018 and August 2023. A total of 65 children (32 boys, 33 girls; mean age 8.9 ± 1.8 years) diagnosed with chronic gastritis at Jun'an Branch Hospital of Shunde Hospital of Guangzhou University of Chinese Medicine between 23 June 2018 and 29 August 2023 were enrolled. The sample size was determined based on comparable diagnostic studies (12).

Inclusion criteria for the chronic gastritis group required:

1. Endoscopic and histopathological confirmation of chronic gastritis;
2. Age 6–12 years.

Exclusion criteria comprised:

1. Severe cardiopulmonary dysfunction or major systemic diseases;
2. History of gastroduodenal surgery;
3. Use of proton pump inhibitors (PPIs)/antibiotics within 1 month or gastroprotective agents within 1 week;
4. Prior *H. pylori* eradication therapy;
5. Any condition contraindicating participation.

For comparative analysis, 50 asymptomatic controls (24 boys, 26 girls; mean age 9.3 ± 1.7 years) were recruited as control group.

Control group inclusion mandated:

1. Age 6–12 years.
2. Absence of gastrointestinal symptoms (abdominal pain, nausea, dyspepsia) for ≥6 months;
3. Normal physical examination and laboratory parameters (complete blood count, hepatic/renal function);
4. Macroscopically normal gastric mucosa confirmed by painless gastroscopy without biopsy (Section 2.2.3).

Identical exclusion criteria were applied to both cohorts.

### 2.2 Methods

#### 2.2.1 Serological test

Serological tests were performed in both groups. Subjects were prevented from food intake after 20:00 the evening before blood collection. The following morning, 5 mL of peripheral blood was collected from the median cubital vein and placed into vacuum tubes. Samples underwent centrifugation at 3,000 rpm for 10 min to isolate serum, which was stored at −80 ℃. Commercial ELISA kits (Biohit Oyj, Finland for pepsinogen; Zhejiang ERKN Biotechnology, China for gastrin) were utilized to quantify serum PG I, PG II, and G-17 levels.

#### 2.2.2 ^13^C-urea breath test

After fasting blood collection, baseline breath samples were obtained. Subjects then ingested a ^13^C-urea capsule (Beijing Boran Pharmaceutical Co., Ltd.) with water. Post-dose breath samples were collected 30 min later. The two samples were analyzed using the HY-IREXA ^13^C breath analyzer (Guangzhou Huayou Mingkang Optoelectronics Co., Ltd.).

#### 2.2.3 Gastroscopy

Gastroscopies were performed in both groups. Control group participants underwent gastroscopy to confirm the absence of gastric pathology. Subjects commenced fasting at 20:00 the evening before the examination. After overnight fasting, painless gastroscopy was performed using an Olympus CV-290 endoscope (Japan). Subjects were anesthetized with intravenous propofol (2 mg/kg). If inflammatory lesions were detected during gastroscopy, 3–5 biopsy samples were collected from the gastric antrum, body, and lesion sites. The collected biopsy samples were immobilized with 37%–40% formaldehyde solution and sent to the pathology department for pathological examination. The biopsy samples were paraffin-embedded, sectioned, and stained with hematoxylin-eosin (H&E) for pathological assessment by certified pathologists. The definitive diagnosis was determined by histological findings.

### 2.3 Statistical analysis

The data were accessed for analysis on 25 September 2023 and statistically processed using SPSS 19.0 software. Quantitative values are expressed as Mean ± SD. One-way ANOVA was employed to compare differences among different groups with data showing normal distribution and homogeneity of variance. Categorical data are displayed as frequency/percentage, and inter-group comparisons were conducted using the *χ*^2^ test. A logistic regression model was developed for multifactorial regression analysis. The predictive accuracy of each test item for chronic gastritis in children was calculated. Diagnostic performance of biomarkers (PG I/II, G-17, ^13^C-UBT) was evaluated via ROC curve analysis. A *P* value of <0.05 was considered statistically significant. All quantitative variables (e.g., PG I, PG II, G-17 serum levels) were analyzed as continuous variables. No grouping or transformation was applied to these variables to preserve their original distribution and maximize statistical power.

## 3 Results

### 3.1 Demographic statistics

This study included 65 children with chronic gastritis (32 boys, 33 girls) and 50 healthy controls (24 boys, 26 girls). All participants underwent a painless gastroscopy under intravenous propofol anesthesia along with three additional gastroenterological examinations. Table 1 shows that there are no statistical differences in baseline demographic characteristics between the two groups.

**Table 1:** Comparison of baseline demographic characteristics between chronic gastritis and control groups.

Group	Number of cases	Age (Mean ± SD)	Male	Female
Control group	50	9.34 ± 1.733	48.00%	52.00%
Chronic gastritis group	65	8.91 ± 1.809	49.23%	50.77%
*P*		>0.05	>0.05	>0.05

### 3.2 Gastroscopic and pathological findings

Painless gastroscopy demonstrated normal results in all 50 healthy controls. On the other hand, all 65 children in the chronic gastritis group were diagnosed with chronic gastritis through gastroscopic and histopathological examinations.

### 3.3 Comparison of positive rates between individual tests and combined detection

**Figure 1:**
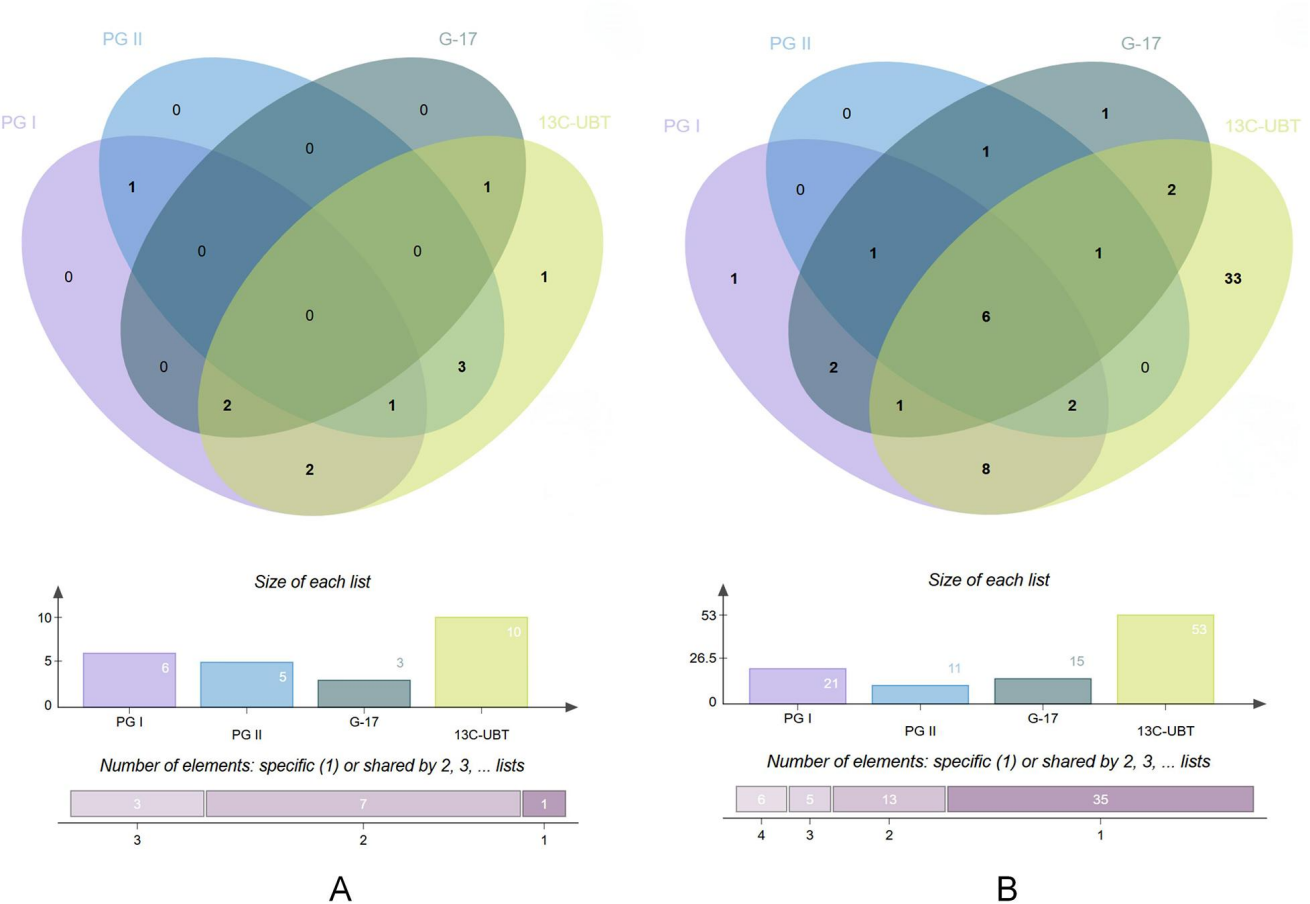
Venn diagrams of the distribution of positive results for PG I, PG II, G-17, 13C-UBT. **(A)** Positive results for the control group; **(B)** Positive results for the gastritis group.

The cut-off values for PG I, PG II and G-17 are 108.10, 13.52 and 8.65, respectively, and are pre-specified thresholds used for binary classification to calculate the positive rate. In the entire cohort, the positive rates for individual tests (PG I, PG II, G-17, ^13^C-UBT) and combined detection (defined as positivity in any of the tests) were 23.48%, 13.91%, 15.65%, 54.78%, and 60.87%, respectively. Compared to the control group, the chronic gastritis group showed significantly higher positive rates for individual test of PG I, G-17 and ^13^C-UBT, and combined detection (*P* < 0.05). The PG II individual test in the chronic gastritis group showed a higher positive rate than in the control group but not statistically significant (Table 2). Figure 1 shows the distribution of positive results for PG I, PG II, G-17 and 13C-UBT between the control group and the gastritis group.

**Table 2:** Comparison of positive rates for PG I, PG Ⅱ, G-17, ^13^C-UBT assays, and combined detection in pediatric chronic gastritis [*n* (%)].

Group	Number of cases	Positive rate for PG I	Positive rate for PG II	Positive rate for G-17 Test	Positive rate for ^13^C-UBT	Positive rate for combined detection
Control group	50	6 (12.00)	5 (10.00)	3 (6.00)	10 (20.00)	11 (22.00)
Chronic gastritis group	65	21 (32.31)	11 (16.92)	15 (23.08)	53 (81.54)	59 (90.77)
*χ* ^2^		6.49	1.13	6.242	43.21	56.11
*P*		0.011	0.288	0.012	<0.001	<0.001

### 3.4 Comparison of serum PG I/II and G-17 levels between groups

PG I, PG II and G-17 levels in chronic gastritis group were significantly higher than those in control group (*P* < 0.05). The calculated PG I/PG II ratio (PGR) was lower in the chronic gastritis group, but the difference lacked statistical significance (Table 3).

**Table 3:** Comparison of PG I, PG II, PGR, and G-17 levels between groups [mean ± SD].

Group	Number of cases	PG I (μg/L)	PG II (μg/L)	PGR	G-17 (pmol/L)
Control group	50	106.78 ± 18.03	16.06 ± 6.03	8.04 ± 1.98	8.04 ± 1.70
Chronic gastritis group	65	116.37 ± 11.53	18.26 ± 5.18	7.94 ± 2.03	9.93 ± 1.78
*P*		<0.05	<0.05	>0.05	<0.05

### 3.5 Multivariate logistic regression analysis of PG Ⅰ/Ⅱ, G-17, and 13c-UBT in predicting chronic gastritis in children

According to the results of multivariate logistic regression analysis, positive levels of PG I, G-17, and ^13^C-UBT were all potential indicators for predicting chronic gastritis in children. PG I substantially impacted the disease, as evidenced by its regression coefficient of 0.046 and a significant *P* value of 0.014 (OR = 1.057, 95% CI: 1.009–1.087). Although PG II had a comparatively high regression coefficient (0.095), there was no statistical significance shown by its *P* value of 0.052. With a substantial impact on the disease (OR = 1.943, 95% CI: 1.348–2.802), G-17 had a *P* value of <0.001. ^13^C-UBT positivity (OR = 17.417, *P* < 0.001) was also a significant predictor of chronic gastritis.

Multivariate logistic regression was used to assess the predictive value of PG I, PG II, G-17, and 13C-UBT for chronic gastritis in children. The regression results are summarized in Table 4. The logistic regression formula is:

$$\begin{array}{rl} \mathrm{Logit}(P)= & \beta 0+\beta 1\times PG\;I+\beta 2\times PG\;II+\beta 3\times G\\ & -17+\beta 4\times 13C-UBT \end{array}$$

**Table 4:** Multivariate logistic regression analysis of PG I/II, G-17, and ^13^C-UBT for pediatric chronic gastritis.

Indicator	Regression coefficient	Standard error	Wald *χ*^2^	*P*-value	OR-value	95% CI
PG I	0.046	0.019	5.983	0.014	1.048	1.009–1.087
PG Ⅱ	0.095	0.049	3.766	0.052	1.100	0.999–1.211
G-17	0.664	0.187	12.652	<0.001	1.943	1.348–2.802
^13^C-UBT	2.857	0.592	23.268	<0.001	17.417	5.454–55.617

Where β0 is the intercept and β1–β4 are the regression coefficients for each predictor. The coefficients and corresponding odds ratios (ORs) are as follows:

PG I: β1 = 0.046, OR = 1.048 (95% CI: 1.009–1.087)
PG II: β2 = 0.095, OR = 1.100 (95% CI: 0.999–1.211)
G-17: β3 = 0.664, OR = 1.943 (95% CI: 1.348–2.802)
13C-UBT: β4 = 2.857, OR = 17.417 (95% CI: 5.454–55.617)

### 3.6 Diagnostic value of PG I/II, G-17, combine blood test and ^13^c-UBT indicators

**Figure 2:**
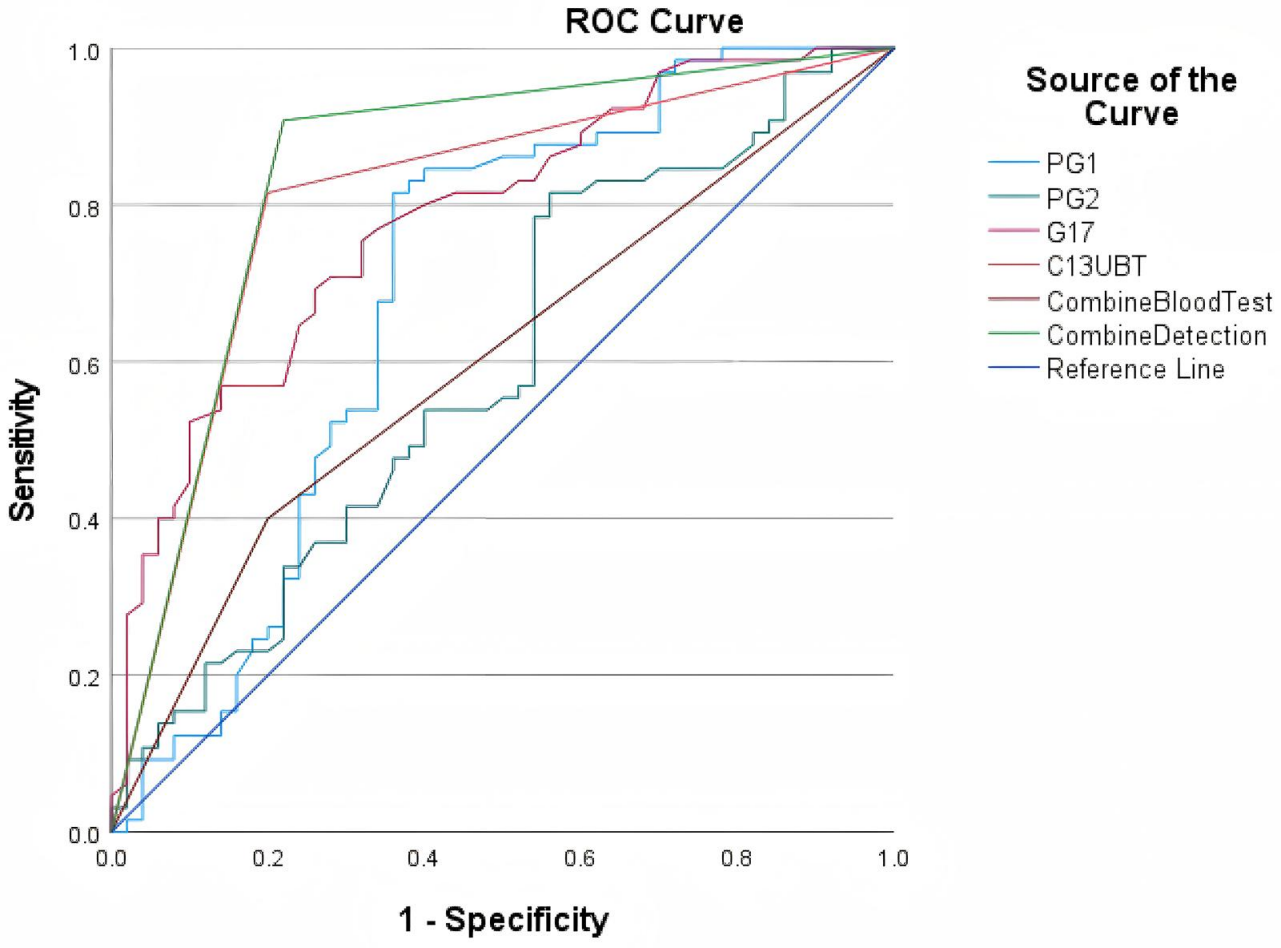
ROC curve analysis.

ROC analysis revealed that the area under the curve (AUC) for PG I, PG II, G-17, and ^13^C-UBT was 0.692 (95%CI: 0.587∼0.796, *P* < 0.01), 0.594 (95%CI: 0.488∼0.700, *P* > 0.05), 0.782 (95%CI: 0.699∼0.865, *P* < 0.01), and 0.803 (95%CI: 0.723∼0.892, *P* < 0.01), respectively (Figure 2). Additionally, the AUC for the Combine Blood Test (defined as a positive result in any of the three hematological tests: PG I, PG II, or G-17) was 0.600, with *P* > 0.05. The combined detection (positivity defined as PG I/II/G-17 exceeding optimal thresholds and 13C-UBT positivity) outperformed the individual tests with an AUC of 0.844, a sensitivity of 90.8%, and a specificity of 78.0%. (Table 5).

**Table 5:** AUC diagnostic value of PG I/II, G-17, ^13^C-UBT, and combined detection.

Indicator	AUC	*P*-value	Optimal threshold	95%CI	Sensitivity	Specificity
PG I	0.692	<0.01	108.10	0.587–0.796	0.815	0.640
PG Ⅱ	0.594	>0.05	13.50	0.488–0.700	0.815	0.440
G-17	0.782	<0.01	8.65	0.699–0.865	0.754	0.680
^13^C-UBT	0.808	<0.01	0.5	0.723–0.892	0.815	0.800
Combined blood test	0.600	>0.05	0.5	0.496–0.704	0.400	0.800
Combined detection	0.844	<0.01	0.5	0.765–0.923	0.908	0.780

### 3.7 Subgroup analysis: Hp-positive vs. hp-negative chronic gastritis

To provide clearer context for interpreting the findings, we stratified the chronic gastritis patients into Hp-positive and Hp-negative subgroups based on their 13C-UBT results, as shown in Table 6. The Hp-positive subgroup exhibited significantly higher serum levels of PG I, PG II, and G-17 compared to the Hp-negative subgroup (*P* < 0.05), indicating that Hp infection is associated with more pronounced alterations in these serological markers.

**Table 6:** Comparison of diagnostic markers between Hp-positive and Hp-negative chronic gastritis subgroups.

Parameter	Hp-positive gastritis (*n* = 53)	Hp-negative gastritis (*n* = 12)	*P*-value
PG I (μg/L)	130.60 ± 14.55	115.35 ± 14.80	<0.05
PG II (μg/L)	21.30 ± 4.90	16.50 ± 5.00	<0.01
G-17 (pmol/L)	11.80 ± 3.20	8.35 ± 1.55	<0.01

## 4 Discussion

The causes of chronic gastritis in children are different from those in adults. Hp infection is a primary causative factor due to their immature immune systems, though dietary habits, environmental influences, and genetic susceptibility also play significant roles (13). The fragile gastric mucosa in children enhances susceptibility to extended inflammation, potentially disrupting gastric acid secretion and resulting in dyspepsia and impaired nutrient absorption. Persistent damage to gastric mucosa may lead to atrophy, elevating the risk of gastric ulcers and other severe gastrointestinal disorders. Chronic gastritis negatively affects the quality of life in children, presenting as recurrent abdominal pain, anorexia, and failure to thrive (14, 15). Early diagnosis and intervention are thus critical to prevent disease progression and long-term sequelae.

Our findings demonstrate that the multi-marker panel (PG I/II + G-17 + ^13^C-UBT) achieved an AUC of 0.844, outperforming individual tests (AUC range: 0.765–0.923), suggesting synergistic diagnostic value. The elevated positive rate of combined testing in the chronic gastritis group, particularly its alignment with ^13^C-UBT outcomes, highlights the critical importance of Hp in the pathogenesis of pediatric gastritisWhile individual biomarkers hold diagnostic value, their synergistic use enhances accuracy and reduces missed diagnoses. Although the Combine Blood Test (PG I, PG II, and G-17) showed a relatively low AUC of 0.600 (*P* > 0.05), indicating a lower diagnostic value compared to other individual tests such as 13C-UBT and G-17, its diagnostic performance could still be relevant in certain clinical scenarios. Further studies with larger sample sizes may help clarify its potential role in the early detection of chronic gastritis in children.

In agreement with previous studies, PG I and PG II levels demonstrate a strong correlation with gastric mucosal dysfunction. Existing literature highlights their utility in monitoring mucosal atrophy, especially during early-stage carcinogenesis (16). Furthermore, G-17, an essential indicator of gastric acid secretion, shows significant variation in individuals with gastric acid secretion disorders. Research indicates that G-17 may possess substantial diagnostic value in chronic gastritis, particularly in pediatric patients with acid-related abnormalities (12). The ^13^C-UBT, extensively used in diagnosing gastrointestinal disorders, validated the association between Hp and chronic gastritis. Routine screening of Hp infection can effectively prevent and cure gastritis and its complications (14). Moreover, combined biomarker assessment provides a holistic evaluation of mucosal health, facilitating early diagnosis (17).

Hp infection is one of the important causes of chronic gastritis. Our subgroup analysis provides a deeper insight into the diagnostic markers. The significant increase in PG I, PG II, and G-17 levels in the Hp-positive subgroup, compared he Hp-negative group, highlights the pivotal role of Hp infection in driving gastric mucosal inflammation and dysfunction in children. This finding is consistent with the established pathophysiology, where Hp infection directly damages epithelial cells, initiates a strong inflammatory response, and disrupts normal gastrin feedback mechanisms (13). We primarily focused on two forms of chronic gastritis: Superficial Gastritis and Helicobacter pylori-associated Gastritis. Superficial Gastritis, characterized by inflammation of the superficial layer of the gastric mucosa, is commonly observed in children and is frequently linked to Hp infection. Similarly, Helicobacter pylori-associated Gastritis is directly caused by Hp, a well-established etiological factor in pediatric gastritis (6). Superficial Gastritis and H. pylori-associated Gastritis are closely related, with the former being an early stage of the latter. If Hp infection is not effectively treated, this superficial inflammation may gradually progress to more severe H. pylori-associated Gastritis and potentially lead to deeper gastric damage.

In our study, although the control group consisted of asymptomatic children, gastroscopy was performed in line with standard clinical practice to ensure the absence of gastric pathology and to confirm the health of the gastric mucosa. This procedure was essential for maintaining the integrity of the control group and ensuring that the comparisons between the chronic gastritis and control groups were valid. No pathological abnormalities were observed in the control group, further confirming their suitability as healthy controls for the study. The decision to perform gastroscopy was based on clinical guidelines and was part of routine medical assessments during the data collection period.

However, certain limitations exist. The limited size and singular focus of the cohort restrict the generalizability of the findings. Results may require multicenter validation due to regional dietary variations. Future research should expand sample sizes and incorporate diverse locations and demographics to improve representativeness. In addition, the higher false positive rate observed for 13C-UBT and combined detection in the control group is similar to the findings of Ramírez-Lázaro MJ (18) (false positive rate of 17% for 13C-UBT, specificity of 83%), which may be attributed to: (1) gastric acid deficiency and dysbiosis in the control group (19), or transient mucosal inflammation caused by dietary or drug stimuli (20); (2) asymptomatic Helicobacter pylori colonization, low-density “occult” Helicobacter pylori infection that cannot be detected by traditional tests, and the absence of histological signs of gastritis (6, 21). Furthermore, due to the distinct pathogenesis of gastritis in children compared to adults, particularly regarding the presentation of Hp infection and the reparative capacity of gastric mucosa, it is crucial to investigate the pathophysiological variations among children of varying ages.

Clinically, early detection of chronic gastritis is critical to prevent irreversible mucosal damage. Non-invasive approaches, such as combined assessment of PG I/II, G-17, and ^13^C-UBT, provide a streamlined diagnostic modality for pediatric gastritis. These serological and breath-based assays demonstrate significantly lower invasiveness than conventional gastroscopy and are particularly suitable for population-level screening in pediatric cohorts. Future research should focus on: (1) validating their utility in monitoring therapeutic responses, and (2) elucidating associations with comorbid gastrointestinal disorders (e.g., peptic ulcer disease, functional dyspepsia). Advances in precision medicine incorporating molecular and genetic biomarkers may further refine diagnostic and therapeutic algorithms. Additionally, screening for virulent *Helicobacter pylori* strains (e.g., Cag A+) in infected children should be prioritized given their established association with gastric carcinogenesis.

## 5 Conclusion

In conclusion, the combined detection of PG I/II, G-17, and 13C-UBT offers robust diagnostic potential for Helicobacter pylori-associated chronic gastritis in children. This multi-marker approach improves diagnostic accuracy and could serve as an effective non-invasive tool for early screening and intervention in pediatric populations.

## Data Availability

The original contributions presented in the study are included in the article/Supplementary Material, further inquiries can be directed to the corresponding authors.
