# Correction: Diagnostic value of combined detection of pepsinogen, gastrin-17, and ^13^C-urea breath test in Helicobacter pylori-associated chronic gastritis in children: a multivariate analysis

**DOI:** 10.3389/fped.2026.1783806

**Published:** 2026-02-11

**Authors:** Yiyun Gao, Chuangui Liu, Kaishi Ouyang, Siqi Li, Weilin Huang

**Affiliations:** 1 Department of Clinical Laboratory, Shunde District Hospital of Integrated Traditional Chinese and Western Medicine (Jun’an Hospital of Shunde District), Foshan, Guangdong, China; 2 Department of Dermatology, Shunde District Hospital of Integrated Traditional Chinese and Western Medicine (Jun’an Hospital of Shunde District), Foshan, Guangdong, China; 3 Department of Anesthesiology, Shunde Hospital of Guangzhou University of Chinese Medicine, Foshan, Guangdong, China

**Keywords:** gastrin-17, pepsinogen, ^13^C-urea breath test, chronic gastritis in children, multivariate logistic regression analysis

**Article type**

The article type of this article was erroneously listed as Review.

This work reports original clinical research findings, including patient enrollment, data collection, multivariate logistic regression analysis, and receiver operating characteristic (ROC) analysis, to evaluate the diagnostic performance of combined biomarkers for chronic gastritis in children. Therefore, the correct article type should be Original Research.

The Data Availability statement was erroneously omitted in the published article. The correct Data Availability statement is “The original contributions presented in the study are included in the article/Supplementary Material, further inquiries can be directed to the corresponding authors.

The original article has been updated.

